# John Faust, V.S.

**Published:** 1902-09

**Authors:** 


### JOHN FAUST, V.S.

The death of John Faust removes from the ranks of the American Veterinary Medical Association one of the few remaining self-made veterinarians who made the early years of the profession rich in progress and honor by their steadfastness of purpose and a rugged honesty of character that made possible the growth and progress of later years.

Those who knew John Faust well and who mingled with him in association circles and in the walks of professional and social intercourse will ever recall the sturdy type of honest manhood that he represented. In the field of discussion his sincerity of purpose was a marked characteristic, and his unbounded sympathies were often expressed by strong emotions that were childlike in their tenderness. While the early trials attending his efforts to obtain a veterinary education were many and such that only a few men would have withstood, he had learned well the lessons along these lines, and planned for the more complete education of his sons by diligence and the toil of his own lifework. This body of men in our profession are fast passing away, but they have done a good work, lived honored lives, and bequeathed to those who have followed them a better field, a stronger profession, and a higher public regard for our worth.

